# Young People’s Experiences of Viewing the Fitspiration Social Media Trend: Qualitative Study

**DOI:** 10.2196/jmir.9156

**Published:** 2018-06-18

**Authors:** Stephanie Easton, Katherine Morton, Zara Tappy, Daniella Francis, Laura Dennison

**Affiliations:** ^1^ Academic Unit of Psychology University of Southampton Southampton United Kingdom

**Keywords:** Social media, Fitspiration, behaviour, health, body image, self-esteem, eating disorders, qualitative research, focus groups, interviews

## Abstract

**Background:**

Social media use has become ubiquitous in the lives of many people, especially young adults. A popular recent trend emerging on social media is that of posting and following ‘Fitspirational’ content - material that purports to motivate and showcase healthy lifestyle habits, particularly relating to exercise and diet. There is very limited existing literature on how engaging with this type of content influences people’s psychological and physical heath. Initial studies have focused on concerns over potential negative effects on psychological wellbeing including body image, self-esteem and eating disorders.

**Objective:**

We aimed to address a gap in the literature for exploratory research on this topic from the perspective of users. We used a qualitative approach to explore how people experience viewing Fitspiration on social media including why and how they engage with this material and how they perceive that it affects their thoughts, emotions, behaviour and health.

**Methods:**

We recruited 20 young adults (14 females, 6 males, aged 18-25) who self-declared themselves to be Fitspiration followers to participate in either focus groups or individual interviews. We asked detailed, open-ended questions about their motivations for following Fitspiration, experiences of viewing this content and its perceived impact. We used inductive thematic analysis to derive themes that represented common and salient features of the data set.

**Results:**

Four main themes were developed: 1) A tool with the potential to support healthy living, 2) Unrealistic, untrustworthy content, 3) Negative effects on emotional wellbeing, and 4) Vulnerability and protective factors. Following Fitspirational posts on social media can provide young people with knowledge and motivation that may support healthy lifestyle behaviours. However, a range of harms also appeared to arise from Fitspiration viewing ranging from minor annoyances and frustrations to more meaningful negative effects on psychological & physical health. These negative effects seemed to persist despite individuals acknowledging that the material can be unrealistic, and believing that they are personally equipped to minimise harms to themselves.

**Conclusions:**

This study suggests that Fitspiration on social media can be attractive and compelling for young people but appears to bring about negative as well as positive effects. Future research should aim to confirm the scale and intensity of positive and negative effects and investigate ways of harnessing desirable outcomes and minimising undesirable outcomes.

## Introduction

In 2017, 66% of UK adults (aged ≥16 years) used the internet for social networking, with 96% of young adults (16-24 years) reported to be active users of social networking sites [1]. Social media platforms enable users to create personal profiles and share content (typically photos and text) and view and comment on the posts of users in their network of friends or “followers” who opt to receive updates on the individual’s social media postings.

In recent years, a trend that has emerged on social media is that of posting and following “Fitspirational” content—material that purports to motivate and promote healthy lifestyle habits, especially associated with exercise and diet. “Fitspiration” is a portmanteau, blending “fitness” and “inspiration” and is particularly prevalent on image-based social media platforms such as Instagram, as well as other platforms, including Facebook, Twitter, Tumblr, and Pinterest. It is essential to be aware that anyone can create a Fitspiration post, just by using the #Fitspiration hashtag. Some posts are created by celebrities and fitness and nutrition professionals, whereas others are created by members of the public who may or may not have relevant knowledge or experience. As of February 2018, the authors identified 14.3 million public posts under the metadata #Fitspiration hashtag on Instagram compared with 1.8 million noted in January 2014 [2], signifying a rapid growth in the popularity of this trend.

Fitspiration emerged within the fitness community as an allegedly healthy alternative in response to previous trends such as “Thinspiration” and “Bonespiration” (images that idealize thin bodies and protruding bones) [3]. Tiggemann and Zaccardo [4] reported that social media content tagged with the “#Fitspiration” hashtag tends to feature people (often females) partaking in exercise or dressed in sportswear, or healthy food; these are occasionally overlain with (ostensibly) inspirational quotes or slogans advocating strength, fitness, personal effort, challenge, and empowerment such as “healthy is sexy,” “eating well is a form of self-respect,” or “be stronger than your excuse.” Some social media accounts attract thousands or millions of followers with some of the most famous posters making money through merchandise and product placement [5]. Social media users encounter Fitspirational content either deliberately, by linking with and following users known for posting such content or inadvertently through the posts and shares of other people already in their network [4].

On the surface, the Fitspiration trend could appear benign or even positive. Efforts to motivate and support young people to develop and maintain healthy lifestyle habits are welcomed and might appear refreshing compared with some more apparently worrying online trends and issues (eg, “Thinspiration”). If people are, indeed, motivated and supported in making healthy lifestyle changes through Fitspiration, the scope for benefits to health outcomes could be massive given the extensive reach of these social media platforms. However, no clear evidence demonstrates these positive effects. While Talbot (2017) determined that Fitspiration does appear to be less objectifying than Thinspiration and Bonespiration, it was still concluded that sufficient similarities existed between these trends to cause concern [3]. Given the newness of the trend, the existing literature remains limited. Several studies using content analysis have concurred that Fitspiration posts perpetuate pervading body image ideals (very lean females and very muscular males), are sexually objectifying, and tend to emphasize appearance over health [6-11]. However, Deighton-Smith and Bell [11] also identified some potentially positive features, including the emphasis on personal control and commitment and building a sense of community and social support. In addition, a concern has been raised about the posters of Fitspirational content, with one study reporting that females who post Fitspirational content have higher scores on measures of disordered eating and compulsive exercise than control females [12]. This study also demonstrated that these females tend to be at higher risk of eating disorders based on scores from the clinical “Drive for Thinness” measure. Furthermore, a content analysis of Fitspirational blogs (in which people write about their experiences of living a healthier lifestyle) provided evidence of problematic eating and negative attitude toward food and body image [13].

Mixed evidence exists regarding how people react to Fitspirational posts. An experimental study reported that exposing female students to Fitspirational images provoked more short-term negative mood and body dissatisfaction and declined appearance-related self-esteem compared with control images [3]. During the study, participants exposed to Fitspiration engaged in more comparison based on their appearance than control participants, and this comparison activity seemingly mediated the effects of the Fitspiration exposure on mood and body dissatisfaction. In addition, participants viewing Fitspiration did exhibit an increase in the measure of inspiration about healthy eating and exercise [4]. A qualitative study interviewed young male Fitspiration followers and revealed that they used the content purposefully (to educate themselves on workout techniques). Although Fitspiration could make them feel inferior about their physical appearance, they engaged in downwards comparison on traits other than appearance and fitness, attributing negative personality traits and neediness to the posters that seemed to help maintain their self-esteem [14].

In summary, although limited research has been conducted to date, the emerging evidence is mixed, suggesting that Fitspiration might exert a negative impact, whereas certain aspects could be inspiring and support behavior change. Thus, further research is warranted on users’ experiences of following Fitspiration. This study used an inductive qualitative approach to address this gap in the literature. Qualitative studies are helpful in emerging fields of research, allowing the emergence of rich data related to a range of perspectives and experiences, rather than constraining data collection to specific anticipated issues [15]. Thus, this study aims to investigate how people experience viewing Fitspiration on social media, including why and how they engage with this material and how they perceive that it affects their thoughts, emotions, behavior, and health.

## Methods

### Design

This study used a qualitative design and collected data using focus groups and individual interviews. We obtained ethical approval from the Ethics Committee of the Psychology Department at the University of Southampton (Southampton, UK; Study ID: 24273).

### Sampling and Recruitment

Participants were eligible for enrollment if they were young adults (aged 18-25 years; as this age group constitutes key consumers of social media, in general [1], and Fitspiration, specifically [16]) who self-defined themselves as followers of Fitspirational content on social media.

Participants were opportunistically enrolled using posters and social media, and course credits were offered to undergraduate students in exchange for participation. In addition, all participants were entered into a prize draw for a chance to win a gift voucher (£15). After viewing adverts, participants contacted the researchers to express interest, read an information sheet, had an opportunity to raise queries, and then signed a consent form before a focus group or interview was arranged.

### Data Collection Procedure

We used focus groups to promote the opportunity for participants to share anecdotes and interact with each other to share experiences and perspectives. Notably, four focus groups were held, which comprised of 4, 2, 5, and 3 participants, respectively. In addition, six individual face-to-face interviews were arranged to accommodate participants who could not attend any of the focus groups.

Each focus group was facilitated by two researchers (SE, ZT, or DF, all psychology students) and began with a welcome statement. Then, each participant completed a short questionnaire covering demographics and brief questions about their social media use, and health-related lifestyle behavior to enable us to define our sample. Participants were shown handouts with a selection of nine examples of Fitspiration to elucidate what is implied by Fitspiration on social media and act as a starter activity to prompt their thoughts on the topic. One example included a picture of a female in activewear with text overlay “how bad do you want it.” A question schedule (Appendix 1) was used to elicit in-depth accounts, stories, or opinions about motivations for viewing/following Fitspirational posts on social media, how Fitspiration is used, aspects that are liked and disliked, and perceptions of the ways in which it might affect their behavior, health, thoughts, and feelings. In addition, we used neutral prompts to probe further and encourage participation from all focus groups’ members. Furthermore, individual interviews followed the same procedure and used the same question schedule as the focus groups.

All interviews and focus groups were audiotaped. The recordings were transcribed verbatim, replacing participant names with pseudonyms. All transcripts were carefully checked against recordings.

### Data Analysis

Using the inductive thematic analysis, we analyzed the transcripts with techniques from grounded theory, such as constant comparison, to ensure the themes being developed remained close to the original data [17,18]. The analysis was led by SE, an undergraduate psychology student and Fitspiration poster and follower, with regular supervision and analytical input from LD, an experienced qualitative researcher and health psychologist, and KM (a trainee health psychologist with qualitative research experience). Of note, the analysis was inductive, that is themes were developed from the participants’ raw data “upwards,” rather than searching for material that fit with a preexisting theory, model, or structure. The analysis started with reading transcripts and listening to audio-recordings to extensively familiarize with the data. Initial coding involved attaching descriptive labels by hand to parts of the transcripts associated with the research question. The analysis proceeded to develop themes from these codes that captured key patterns and features in the data. Then, the theme development was attained by an iterative process of clustering together similar codes into themes while engaging in the process of constant comparison with the original transcripts to check that themes were grounded in the data and were not being affected by the researchers’ preconceptions or theoretical assumptions [18]. Finally, themes were iteratively reviewed, refined, organized, and relabeled until a set of rich, coherent themes, and subthemes was created in a coding manual (Appendix 2).

In line with the grounded theory approach, analysis and data collection were performed concurrently, enabling us to (1) adjust our sampling strategy to deliberately sample participants with characteristics that were underrepresented in our sample; and (2) adjust the data collection to follow up on analytic insights and emerging ideas [19]. For example, an early analysis of transcripts suggested some possible differences in how males perceive Fitspiration; however, we only had limited male participants. Thus, we sought out more male participants to collect more data to explore this insight further. We ceased recruitment after data had been collected from 20 participants, as it became apparent that significant repetition was occurring, adding little new insight to the ongoing analysis.

## Results

### Participants

In this study, we enrolled 20 people (14/20, 70%, females and 6/20, 30%, males; age range: 18-25 years; mean age: 20.7 years). Table 1 summarizes the characteristics of the study cohort. The majority of participants (14/20, 70%) were white-British, and 16/20 (80%) were students, including 14 undergraduates and 2 postgraduates.

Of all, 14/20 (70%) reported spending a minimum of 2 hours on social media each day, with 9/20 (45%) spending >1 hour per day on health and fitness-related content, and a minority 1/20 (5%) spending >4 hours per day. The most popular social media platforms for health- and fitness-related content were Instagram, Facebook, and YouTube.

**Table 1:** Participant characteristics.

Characteristic			Value
**Demographics**			
	Age (years), mean (SD); range		20.7 (1.79); 18–25
	**Gender, n (%)**		
		Female	14 (70)
		Male	6 (30)
	**Occupation n (%)**		
		Undergraduate student	14 (70)
		Postgraduate	2 (10)
		Non-student	4 (20)
		Ethnicity	
		White-British	14 (70)
		Black-African	1 (5)
		Black-Caribbean	1 (5)
		Asian-Indian	1 (5)
		Other	3 (15)
**Social media use, n (%)**			
	**Daily hours spent on social media^a^**		
		<1	2 (10)
		1-2	4 (20)
		2-3	10 (50)
		4+	4 (20)
	**Daily hours spent on health- and fitness-related social media^a^**		
		<1	11 (55)
		1-2	7 (35)
		2-3	1 (5)
		4+	1 (5)
	**Social media sites frequently used to view health and fitness related content^a^**		
		Facebook	11 (55)
		Twitter	1 (5)
		Instagram	18 (90)
		Snapchat	5 (25)
		Pinterest	2 (10)
		YouTube	11 (55)
		WordPress	1 (5)
		Other	2 (10)
**Perceptions of health and lifestyle, n (%)**			
	**Which would you consider yourself to be?^a^**		
		“Underweight”	1 (5)
		“About right”	18 (90)
		“Overweight”	1 (5)
	**Would you say you lead an active lifestyle?^a^**		
		Yes	17 (85)
		No	3 (15)
	**Would you say you eat a healthy diet?^a^**		
		Yes	17 (85)
		No	3 (15)

^a^Self-reported estimates.

**Table 2:** Themes and subthemes.

Theme	Subtheme
A tool with some potential to support behavior change	Information and ideas Being inspired and motivated
Unrealistic, untrustworthy content	Trust and deception Unrealistic unattainable lifestyles Inappropriate or abandoned goals
Negative effects on emotional well-being	Feeling guilty about choices and behavior Feeling low about my body Concerns about eating Feeling compelled to keep using Fitspiration
Vulnerability and protective factors	Gender Age Mood Engaging in a critical way Filtering and choosing relevant content to follow

Furthermore, 18/20 (90%) participants classified themselves as being of healthy weight, 17/20 (85%) felt they led an active lifestyle and 17/20 (85%) ate a healthy diet.

### Themes

We developed the following four key themes in this study: (1) *a tool with some potential to support behavior change*; (2) *unrealistic, untrustworthy content;* (3) *negative effects on emotional well-being*; and (4) *vulnerability and protective factors*. Table 2 presents these themes and their subthemes; also, each theme and subtheme is discussed alongside illustrative quotations in the following section. We have replaced participant names with participant numbers, and gender and age have also been indicated (eg, P1, F, 20).

### A Tool With Some Potential to Support Behavior Change

Participants revealed benefits that could be gained from Fitspiration content that facilitated making changes to their behavior.

#### Information and Ideas

Participants discussed following Fitspiration accounts on social media to gain practical ideas and tips about healthy lifestyles. They discussed how Fitspiration content successfully provided them with ideas for healthy recipes, workouts, exercise techniques, and gym merchandise.

It is nice to see to get some ideas […] when they post exercises and I think “oh that might be something I haven’t tried before.”

P1, F, 20

#### Being Inspired and Motivated

Participants described how Fitspiration content boosted their motivation to attend a gym, follow a nutritious diet, and helped them to adopt a positive mind-set. They described how motivation could be explicitly triggered by written, inspirational quotes.

It helps me to set targets [...] see what I need to be doing and then kind of get me the road to doing it.

P3, F, 19

In addition, observing posters helped them attain their goals and boosted motivation for working toward their health targets, and individuals posting Fitspiration content acted as aspirational figures and role models.

I think they can be good for getting you motivated like definitely, […], if I try hard I could look like this.

P5, F, 20

### Unrealistic, Untrustworthy Content

All participants discussed how they often find Fitspirational content to be unrealistic and difficult to associate with. Besides creating frustration and negative feelings toward Fitspiration posters, the unrealistic content seemed to adversely affect their goal setting and perseverance.

#### Trust and Deception

Some participants explained the difficulty in determining which information could be trusted. With no evidence of qualifications, many participants were uncertain which Fitspiration posters possessed adequate expertise to offer valid advice. In addition, participants were conscious that Fitspiration posters mislead and deceive users with filtered content, good lighting and specific poses, and cherry-picked only the best parts of their lives to share.

People are putting up their best photos for a reason, and it’s not like real life

P2, F, 21

Participants were concerned about Fitspiration posters having ulterior motives for posting material, especially an awareness that some posters might gain financially by supporting and endorsing brands.

They might have some like agenda, and maybe they’re not being so honest about that

P14, F, 25

Furthermore, participants discussed various types of products they had seen endorsed or advertised and conveyed frustration that they were being sold products rather than being offered valuable advice. Seemingly, they found it difficult to distinguish whether some Fitspiration posts had hidden intentions to promote products shown in the post, making them wary of trusting the content of the post.

They are just getting money out of it […] that is all they are doing it for.

P1, F, 20

#### Unrealistic Unattainable Lifestyles

Participants perceived that the lifestyles depicted by many posters were difficult or impossible to associate with and emulate. More famous posters were perceived to possess luxuries that suggested an advantage over their followers, making the lifestyle less obtainable.

You have a gym in your house! How is that like real life?

P6, F, 20

In particular, participants were especially distrustful of celebrities, as they felt that cosmetic surgery and body-alteration made them unrealistic models for followers.

They’ve all had surgery so people will be working towards a goal that’s not achievable

P19, F, 22

Several participants (whether students or employed) considered cost as a barrier to living a healthy lifestyle, especially because of the perceived cost of the food, gym memberships, and clothing.

I think something that’s not taken into account is that being fit is like money as well, like gym membership and gym clothes and healthy food, that’s so much money so if you can’t afford it you feel like, well I feel like there’s no point.

P9, F, 18

In addition, participants believed they lacked time to adhere to the lifestyle presented, with one participant even describing poster’s habits to be a full-time job.

A lot of people I follow are quite into it and sort of do it every day and most of the time that’s all they do […] you don’t really know whether, if they have another job or not.

P16, M, 22

#### Inappropriate or Abandoned Goals

Several participants discussed how Fitspiration, though recognized as untrustworthy and unrealistic, could still affect the types of goals they were aiming for and made them less attainable.

It’s probably made my personal goals quite different because they [the Fitspiration figures] obviously look amazing… I wouldn’t have set goals that unrealistic if I didn’t follow them on social media.

P15, F, 21

It was recognized that this perspective was associated with feelings of disappointment and pressure.

They [the posters] put…’you can achieve this in 6 weeks’…and it’s physically impossible to achieve that kind of physique in that amount of time and I feel it puts an unfair pressure on.

P18, M, 24

Some participants reflected that unrealistic content resulted in disengagement with goals that were overly ambitious.

It can make you give up quicker I think

P11, F, 19

In addition, participants commented that following Fitspiration made their goals more focused on appearance and gaining approval from peers, rather than health.

You kind of lose sight of the goal of actually trying to become healthy rather than just looking good for pictures on social media.

P16, M, 22

In fact, one of the participants perceived this focus on image over health as an issue with Fitspiration posters, implying that they have the wrong priorities.

They don’t go to the gym and things because they want to be healthy and lead a healthy lifestyle, they want to have a body from which they can take pictures and post it to Instagram.

P16, M, 22

### Negative Effects on Emotional Well-Being

Participants discussed various negative emotional experiences stemming from viewing Fitspiration content. Mostly, this discussion was about personal experiences and feelings, whereas other comments seemed more speculative and hypothetical.

#### Feeling Guilty About Choices and Behavior.

One of the most often discussed feelings was guilt about not following a similar lifestyle to those advocated in Fitspiration posts. Viewing Fitspiration posts seemingly provoked participants to compare these with their health and fitness-related habits and feel guilty when they did not match up.

It makes me feel quite guilty sometimes, if you’re just not really in the mood to um, like be productive or proactive […] and then you see all these posts, and it’s telling you that you should.

P13, F, 20

In fact, some participants displayed ambivalence around this guilt response as they knew that guilt was not appropriate or logical because of their awareness of the unrealistic nature of the posts.

I feel so guilty if I see all this, but then I’m like why am I feeling guilty? Because what I have just done is normal.

P1, F, 20

#### Feeling Low About My Body

Participants reported being left with negative feelings about their body when comparing themselves with Fitspirational images.

It makes me not enjoy things like going to the beach and like taking photos on holiday because you don’t look like the photos on Instagram.

P15, F, 21

When I see fitness accounts where all the girls are like svelte and toned, I’m like oh, it’s hard to love me when I look like this.

P6, F, 20

In fact, one participant highlighted that those who differ from the typical body type within Fitspiration content could be at a heightened risk of experiencing these negative feelings.

If you’re of a bigger size, it can make you feel horrendous, it can make you feel completely alien and that you shouldn’t look like that.

P15, F, 21

Moreover, failure to make rapid progress toward the ambitious appearance-related goals that they had set for themselves could trigger negative feelings about themselves.

You can’t have this tiny waist and massive bum, […] you may if you did it [exercise] for a few years, a long time, […] but it can make you feel kinda down about yourself.

P8, F, 20

#### Concerns About Eating

Participants indicated that Fitspiration exerted both positive and negative effects on their eating habits. Although increased awareness about food choices was described by some participants, others found following the eating plans advocated by posters impossible to sustain and were aware of rebounding to extremes of unhealthy eating, or even binge eating.

I’m a lot more aware of food groups, the whole ideal food groups plate arrangement, it’s like half vegetables, a quarter of protein, a quarter of carbs, I’m very aware of doing that when I have my dinners.

P6, F, 20

I think it has made me a lot more wary of what I put into my body but then I will have blow out days and just like literally shove food down.

P5, F, 20

Some other participants discussed that viewing Fitspiration posts encouraged their obsession with calorie counting. In fact, a few also believed that some of the diet-related material could even instigate an eating disorder, especially if they were unable to recognize that habits were becoming unhealthy.

If I followed their food account where they tell me to eat healthily and I couldn’t, I’d probably end up with an eating disorder.

P6, F, 20

#### Feeling Compelled to Keep Using Fitspiration

Several participants described experiencing conflict as they knew that Fitspiration posts could elicit various negative thoughts, behaviors, and moods; yet, they found themselves viewing it regularly.

In one way you’re like really attracted to it but in some ways you find it really annoying and it puts you down

P14, F, 25

In addition, some participants described how they had initially followed Fitspiration content for a specific purpose and believed that it had not successfully fulfilled that purpose, but having got involved in the social and community aspect of it they felt compelled to continue engaging with it. Furthermore, many seemed to find this type of social media usage compelling or even addictive.

### Vulnerability and Protective Factors

Participants perceived that various contextual factors affected the degree to which they and others experienced negative impacts from Fitspiration content.

#### Gender

Several participants (both males and females) believed that females tended to be more vulnerable to the negative effects of the exposure of Fitspiration and, indeed, mass media more generally than males.

Females tend to be more sensitive […] it can have a bit more of a deeper effect on them, whereas men tend to be a bit more hard-headed.

P16, M, 22

In line with this, most of the talk about guilt, body image, and concerns about eating and compulsive viewing came from females (refer “Negative Effects on Emotional Well-being” theme above). In addition, participants perceived that Fitspiration perpetuated a long-standing pressure to conform to the existing female body ideals.

For years there’s been this problem with media, especially girls like feeling they need to look a certain way.

P8, F, 20

Yet, one male participant described negative emotions associated with failing to fulfill appearance-based expectations that had been generated by following Fitspiration posts.

I think mentally, it’s quite stressful sometimes if you put yourself up to a task that you can’t achieve […] looking at yourself in the mirror […] you’re just not really seeing results, it can definitely have a negative impact

P16, M, 22

Furthermore, the participants speculated that males may well be negatively affected but might not express their feelings because of gender norms.

I reckon it probably negatively affects boys, but they don’t express it […] If a boy did he’d probably be called a wimp.

P6, F, 20

#### Age

Participants anticipated that younger users than themselves tended to be deceived by unrealistic content, consumed by the lifestyle and, thus, most likely to experience negative effects.

In line with this, one participant suggested that her maturity enabled her to control how much the content affected her behavior.

I am old enough, wise enough to know that it is cool too if I have had Uni all day [and therefore not had time to exercise] then that’s fine.

P2, F, 21

#### Mood

Participants discussed their affective state influencing how they responded to Fitspiration posts and elucidated how Fitspiration could intensify their emotional state if they were in a bad mood. Conversely, if they were viewing Fitspiration while already in a good mood, it seemed to buffer against negative effects and the material could enhance their motivation to emulate healthy behaviors they have witnessed online.

Let’s say I’m already feeling up for some workout […] then I see some Fitspiration post, I might be like more inclined to go and do it and then feel more like, positive about it but if I’m in a bad mood then I don’t want to see that, I just get grumpy

P14, F, 25

More worryingly, participants discussed how distress associated with life events, such as the end of a relationship, could render them highly sensitive to negative effects of Fitspiration content.

Combined with just being broken up with, it just like destroyed my self-esteem seeing all these really fit people.

P5, F, 20

#### Engaging in a Critical Way

Seemingly, participants adopted certain approaches to Fitspiration viewing that they believed enabled them to follow this content with a decreased risk of psychological harm. One technique was to use the content purposefully but step back from getting too immersed.

It’s kind of best to keep them at a distance […] use them for inspiration now and then but I don’t think it’s healthy to be completely immersed

P1, F, 20

Others, however, felt they had adequate knowledge or education that enabled them to assess the messages and images being presented critically.

I kind of have got the knowledge to know that your abs just aren’t going to just appear

P1, F, 20

Moreover, some were especially aware and well-informed about tricks and effects of the media and believed that their less naïve and more critical approach provided them with some protection against negative effects of Fitspiration.

I study marketing […] I’m a lot more knowledgeable and less naïveto the content being advertised to me.

P19, F, 22

However, participants who felt their knowledge protected them still provided accounts of various ways that Fitspiration viewing had adversely affected them personally.

#### Filtering and Choosing Relevant Content to Follow

Some participants carefully selected and filtered the content that they followed and engaged with to get the most from it. For example, a few participants described selecting content to view that was consistent with their goals and minimizing exposure to material perceived as irrelevant.

When they put up their personal life and things…I’m not interested in them as a person which is probably quite bad but I just want to see the videos of what they do in the gym.

P15, F, 21

Furthermore, several participants discussed being selective in following Fitspiration posters who they felt they could associate with to ensure their goals were more realistic to attain.

I always like to follow normal people as well […] these things are actually achievable.

P19, F, 22

## Discussion

### Key Findings

This in-depth qualitative study exploring the experiences and perspectives of Fitspiration followers revealed several crucial insights. Consistent with Palmer [14], this study reported that participants described a desire to gain information as a critical driver of consumption of Fitspirational material; specifically, our participants were interested in gaining information related to exercise techniques, healthy recipes, and workouts. Like Tiggeman and Zaccardo [7], this study demonstrated that participants felt inspired and motivated by Fitspirational content; however, this does not seem to translate into positive dietary change and physical activity routinely, a finding also reported by Palmer [14]. Nevertheless, the majority of our findings highlighted concerning aspects of the Fitspiration use. Our participants discussed several negative effects ranging from minor (eg, frustration about the deceptive nature of posts, jealousy regarding unattainable body appearance or lifestyles, feeling that their usage had become out of control, guilt about not following the lifestyles advocated, and frustration in being encouraged toward inappropriate goal-setting) to more disconcerting (eg, negative feelings toward their own bodies and indications of some concerning eating habits). The issues of frustration, guilt, and feeling addicted to viewing Fitspirational posts are novel insights emerging from this study. Previously, negative effects of the exposure to Fitspiration on self-esteem, body image, and disordered eating have been reported [4] and speculated [6-13]; this study is in line with these studies and also the proposition that social comparison based on appearance is one of the routes through which Fitspiration exerts negative psychological effects [4].

We determined that our participants were critical, cautious, and questioning of Fitspirational content, highly aware of authenticity and credibility issues, and some made mindful decisions about who and what to follow and what aspects of posts to focus on, which corroborated Palmer’s [14] study. In our study, some participants felt that their age, gender, education, or approach to using Fitspiration protected them from a negative psychological impact. However, negative psychological effects seemed to persist despite participants possessing characteristics and capabilities that they believed could buffer them against harm. In addition, our participants exhibited a considerable conflict and ambivalence around Fitspiration. They persisted in viewing content despite feeling it could be frustrating or even harmful. They also reported feeling guilty and comparing themselves unfavorably to posters despite articulating how guilt is unwarranted as posters are showing edited versions of themselves and sending invitations to lead unviable lifestyles and fulfill impossible appearance and health-related goals. Finally, participants discussed female gender as a factor associated with vulnerability to negative effects from using Fitspiration; however, this finding might have been driven by stereotypes and gender norms. When reviewing males’ descriptions of impact, this study provided evidence that males, as well as females, could be adversely affected by content and were similar in the ways they thought and felt about the material they were seeing.

### Study Strengths and Limitations

This study has several strengths and limitations that merit consideration while interpreting the study findings. First, sample composition. Our sample size was relatively small (*n*=20). However, we attained saturation with this number of participants. In addition, we successfully sampled participants with various views and experiences. We attained a variability in age, gender, and ethnicity and enrolled participants that varied in their intensity of engagement with social media, generally, and Fitspiration, specifically. Furthermore, the sample comprised participants who reported being both committed and uncommitted to healthy eating and exercise, although the majority reported being committed. Most participants perceived their weight as “about right,” although it is essential to note that the questionnaire only captured self-reported perceptions of weight and not actual body mass index (BMI). Being an exploratory qualitative study, we did not assess participants’ objective weight, although this could be an exciting avenue for further research on the impact of Fitspiration.

University students accounted for the majority of our sample. Thus, the findings of this study represent views and experiences of a highly educated group of young people. In addition, since our recruitment strategy resulted in the inclusion of a high number of psychology students, our participants may, because of their education, have been especially attuned to the negative effects of the media and possibly more likely to consider and discuss issues associated with eating disorders and body image. Nonetheless, while some of the discussion was speculative and hypothetical, there was also a considerable discussion of personal experiences and feelings. Overall, because of our sampling, we suspect that our participants might have been more critical and careful with their social media use and plausibly better protected against the negative impact than other young people who follow Fitspiration.

Second, another methodological consideration associated with data collection. The use of focus groups maximized opportunities to create discussion and enable participants to draw on, compare, and contrast experiences and perspectives. However, perhaps social desirability, gender norms, or the effect of dominant individuals could have silenced dissenting opinions and rendered it more difficult to talk about specific personal experiences. In addition, participants might have found that having young female students as interviewers and facilitators made them reluctant to mention specific issues. We did, however, determine that several participants discussed emotive and personal experiences, and we were reassured that individual interviews elicited similar issues to those raised in focus groups.

Finally, it is imperative to consider that participants might have had concerns about their body image and eating habits before their exposure to Fitspiration. From this study, it is not possible to understand to what extent Fitspiration causes these negative outcomes. However, it remains interesting that participants perceive that Fitspiration is accountable for these feelings.

### Future Research

This study suggests several useful directions for future Fitspiration research. Experimental, quasi-experimental, or observational studies could be conducted to attain a quantitative, more objective assessment of the factors emerging as important from this exploratory qualitative study [20,21], including short- and long-term emotional, cognitive, and behavioral responses to Fitspirational content. This study suggests that frustration, guilt, compulsive social media use, self-esteem, body image, and concerns with eating are relevant negative outcomes to measure and that health- and fitness-related knowledge, motivation, and behavior change are relevant positive outcomes to measure. In addition, this study points toward the utility of examining possible moderators of the impact of Fitspiration, including the realism and relatability of posters and the posted material and the characteristics of the follower, including age, gender, preexposure mood, and media-related literacy and critical appraisal skills. This study supports previous suggestions that social comparison could be investigated as one potential mediator of negative effects on the body image and related outcomes [3]. Furthermore, future studies could consider focusing on different groups of Fitspiration followers. A slightly younger (ie, teenage) group might have different motivations for following Fitspiration. However, this age group has been neglected in Fitspiration research thus far.

Given that the emerging research literature on Fitspiration has indicated potential harm yet statistics demonstrate that Fitspiration is a growing trend, it seems useful to consider interventions to decrease or prevent harm from body and appearance-related images and content on social media. Previously, some studies have suggested that psycho-educational interventions could focus on raising awareness of the harms of this sort of content and increasing followers’ media literacy and critical appraisal skills [22,23]. This study suggests young people believe that being armed with information and critical appraisal skills will protect them from possible negative effects. However, this study also suggests that critical and careful consumption of Fitspiration might not actually be successful in averting the negative psychological impact. Thus, further exploration and evaluation of potential intervention approaches is warranted.

An alternative direction for research is capitalizing on the positive aspects of Fitspiration in social media. Researchers could develop or adapt theory-based behavior change interventions to enhance the diet and physical activity levels of young adults, which make use of social media platforms to deliver appropriate health-related messages [24]; these platforms are appealing, influential, and draw in young females in particular [16]. A key challenge could be mimicking what is appealing, engaging, and positive about Fitspiration while omitting what seems harmful.

### Conclusions

This study suggests that following Fitspirational posts on social media can provide young people with knowledge and motivation to support healthy lifestyle behaviors; however, following such content also seems to exert some undesirable effects. In addition, this study suggests the possibility of various harms ranging from minor annoyances and frustrations to more meaningful effects on the mental and physical health. These effects might persist despite users being aware of how unrealistic the material portrayed can be and despite users believing that they are personally well-equipped to minimize harms to themselves. Furthermore, studies to confirm benefits and harms and investigate ways of harnessing positives and minimizing negatives would be helpful additions to this field of research.

## Appendix

Multimedia Appendix 1 Interview schedule.

Multimedia Appendix 2 Coding manual.
