# Supplementary material for: Young People’s Experiences of Viewing the Fitspiration Social Media Trend: Qualitative Study
Source: J Med Internet Res. 2018 Jun 18;20(6):e219. doi: 10.2196/jmir.9156 (PMC6028764; doi:10.2196/jmir.9156)
Supplement: Multimedia Appendix 1 [file jmir_v20i6e219_app1.pdf]

Appendix 1. Interview Schedule

Can you begin by telling us what you know about the trend of ‘Fitspiration’ on social media?
Tell us about how you first came to encounter social media ‘Fitspiration’.
Can you tell us about how you came to follow this sort of content on social media?
Tell us about what type of health and fitness broadcasters you follow.
Prompts might include:

a. Professional or non-professionals
b. Social media starts? Celebrities? Dietitians? Personal Trainers?

Can you describe the type of content/posters that you tend to see?
Can you tell us about anything you like about Fitspiration page/posts?
Can you tell us anything you dislike about Fitspiration pages/posts?
Can you describe anything that makes you feel good on Fitspiration pages/posts?
Is there anything that makes you feel bad on Fitspiration pages/posts?
Can you tell me about any ways that you think this type of social media content affects you?
Prompts might include:

a. Tell us about whether there are any ways that it affects your behaviour (your habits, the things you do)
b. Can you describe whether there are any ways that it impacts on your health?
c. Can you tell us any ways that it influences how you think or feel?
d. Is there any other way that is affects you?

General Prompts:
Can you tell me more about that?
Can you expand on that?
Can you think of an example?
How did/do you feel about that?
What’s that like for you?
