# Supplementary material for: Young People’s Experiences of Viewing the Fitspiration Social Media Trend: Qualitative Study
Source: J Med Internet Res. 2018 Jun 18;20(6):e219. doi: 10.2196/jmir.9156 (PMC6028764; doi:10.2196/jmir.9156)
Supplement: Multimedia Appendix 2 [file jmir_v20i6e219_app2.pdf]

Theme	Subtheme	Participant	Quote examples
**A tool with some potential to support behaviour change**	Information and ideas	P1, F, 20	*“Sometimes it is nice to see to get some ideas as well… when they post exercises and I think oh that might be something I haven't tried before just to do something different”*
		P3, F, 19	*“It’s helpful to watch others do exercises”*
		P15, F, 21	*“I started going to the gym and wanted to get a more varied idea of what I could do in the gym, so I started looking on different sites and found lots of things, Instagram in particular, of people that had put videos up of how to do things”*
	Being inspired and motivated	P1, F, 20	*“Have a look into their plans just by seeing their cooking and exercises that they are doing and you can kind of get a little bit of inspiration from it without having to spend £60 on it”*
		P3, F, 19	*“I feel it's good because it helps me to set targets and it helps me to see what I need to be doing and then kind of get me the road to doing it”*
		P5, F, 20	*“I think they can be good for getting you motivated like definitely, and like you do thing ah this is pretty cool, like if I try hard I could look like this”*
		P11, F, 19	*“I think it helps you get into a routine as well, you see them every day, come up on your feed every day, you'll kinda get set into a routine of going to the gym and maybe after a few weeks then it kinda more set in your habits”*
**Unrealistic, untrustworthy content**	Trust and deception	P6, F, 20	*“They spout balance but don't really mean it, like [name] profile is always like it’s all about balance" but it's like you have a gym in your house, like how is that like real life?”*
		P8, F, 20	*“The only other thing I think is like sometimes and this goes back to like saying quite a lot of the images are quite deceiving I think they like quite often show people that like you expect if you do these exercises your body is going to look like that”*
		P11, F, 19	*“Those pictures are deceiving a lot of the time, like just the way they position their body”*
		P14, F, 25	*“They might have some like agenda and maybe they're not being so honest about that”*
	Unrealistic, unattainable lifestyles	P2, F, 21	*“Moneywise it is expensive if you have gym memberships and they spend a lot on their gym clothing and to meal as well that is a lot of…and especially if you are following celebrities they get sent like food and all the stuff”*
		P6, F, 20	*“Cause realistically at university you can't afford half the stuff they eat, like that Gwyneth Paltrow's diet”*
		P12, F, 21	*“It might look really nice but some of the ingredients like chia seeds or this powder or whatever are like, it's just quite an expensive way to cook”*

		P16, M, 22	*"When it comes to the realism of having to work during the week and the limited time that I get to put towards it then it takes a swing and makes you feel quite down about it because you can't put in the same amount of time and effort"*
	Inappropriate or abandoned goals	P15, F, 21	*"It's probably made my personal goals quite different because they [the Fitspiration posters] obviously look amazing…I wouldn't have set goals that unrealistic if I didn't follow them on social media"*
		P16, M, 22	*"You kind of lose sight of the goal of actually trying to become healthy rather than just looking good for pictures on social media"*
		P18, M, 24	*"They [the posters] put 'you can achieve this in 6 weeks'…and it's physically impossible to achieve that kind of physique in that amount of time and I feel it puts an unfair pressure on"*
		P11, F, 19	*"It can make you give up quicker I think"*
**Negative effects emotional wellbeing**	Feeling guilty about choices and behaviour	P1, F, 20	*"They can make me feel like guilty sometimes and I need to take a step back and think actually I don't need to feel guilty but it's just an automatic response when you see people who have eaten all this healthy stuff in the day, done all this exercise and then you are like ooh I haven't really done that but then you're not them, you're doing what is good for you so...but I think it is an automatic response, for me anyway, I feel guilt straight away"*
		P6, F, 20	*"At the same time, even though you're recognising you're being lied to, you still can't help but feel you'll, like you'll never be good enough…to get to that point, like you'll never realistically get to that point, that's a bit disheartening"*
		P13, F, 20	*"It makes me feel quite guilty sometimes, if you're just not really in the mood to um, like be productive or proactive and get out of bed and go on a run or go to the gym or something and then you see all these posts and it's telling you that you should"*
		P15, F, 21	*"When I've eaten really badly or - and I've eaten all the food in the world that day and I realise I'm not going to look like them if I carry on like that it makes me feel quite bad"*
	Feeling low about my body	P8, F, 20	*"If I didn't do exercise, would I be thinking well I'm not healthy does that mean I'm not sexy?"*
		P15, F, 21	*"I think it affects me when I'm going on holiday and I don't look in a bikini how they look and it makes me not enjoy things like going to the beach and like taking photos on holiday because you don't look like the photos on Instagram"*

		P8, F, 20	*Those kind of things I think subconsciously play a role in how you feel about yourself, if you constantly see that kind of thing about like you're only attractive if you do this kind of thing, I think it can be quite difficult maybe…you can see that kind of thing on a regular basis will have some effects subconsciously"*
	Concerns about eating	P5, F, 20	*"Food pictures come up [on Instagram] and then people put the like calories and I'm a massive calorie counter…every bite I eat that I put into my body, it is horrendous"*
		P6, F, 20	*"If I followed their food account where they tell me to eat healthily and I couldn't, I'd probably end up with an eating disorder"*
	Feeling compelled to keep using Fitspiration	P5, F, 20	*"When you start following one, it just leads you to like more"*
		P14, F, 25	*"In one way you're like really attracted to it but in some ways you find it really annoying and it puts you down"*
		P11, F, 19	*"I think it becomes obsessive, maybe people are obsessed with the certain image and maybe wanting to look a certain way, so then they follow all these accounts and like aim to kinda look the way they want to look"*
**Vulnerability and protective factors**	Gender	P8, F, 20	*"I think for guys it might be slightly less bothered about what's on social media and more bothered about what they actually see"*
		P11, F, 19	*"You probably think if affects females more but realistically it wouldn't…that's the kind stereotypical view that women are more sensitive and care about their bodies more when actually it's not quite accurate"*
		P14, F, 25	*"I don't think men talk about it that much although my boyfriend does that "I need to get more ripped" and whatever and I do think he definitely has like sometimes if like, when looking at pictures, what's the male ideal body type? And I'll be like showing less muscular than for him for example and he's like "he really needs to be ripped" so yeah maybe it affects everyone"*
		P16, M, 22	*"I feel like females are more superficial with it than males and I feel like they tend to be the people who care more about social media and posting social media pictures and are perhaps not as committed because when you go into a gym, it will tend to be more male dominated"*
		P16, M, 22	*"I think women are potentially more likely to get involved and drawn in through the fitspiration on social media and it's more likely to have negative impact on them, whereas men are probably more likely to go down the traditional route of actually, um, doing it for more - more health benefits than just superficial nature"*
	Mood	P7, M, 19	*"I find that it comes in short little bursts, there's a week*

			when I'm really motivated and there is another week when I'm like binge-eating chocolate and watching videos"
		P8, F, 20	*"It depends on what's going on at the time, like if I'm in a position where I'm feeling generally really positive, it's sunny outside or nearly summer and then I'm like really up for going to the gym and I think I'm going to look like her and I'm going to be really good and then it's really very positive and other times I'm just like uhhh no"*
		P14, F, 25	*"Let's say I'm already feeling up for some workout or like I know I should go and I then I see some fitspiration post, I might be like more inclined to go and do it and then feel more like, positive about it but if I'm in a bad mood then I don't want to see that, I just get grumpy"*
	Age	P1, F, 20	*"If there is younger people following them then it can do more harm than good"*
		P1, F, 20	*"Yeah especially for young teenagers, erm yeah I can imagine they can like, coz it's something for them to look up to and they take everything literally"*
		P2, F, 21	*"I think young people would listen"*
	Engaging in a critical way	P1, F, 20	*"I have realised I see all these people but I can take a step back and I realise that's not realistic and I'm doing that is right for me and if I have a day of eating chocolate then I don't punish myself for it"*
		P2, F, 21	*"Well I try and sort of remember that on Instagram, people are putting up their best photos for a reason and it's not like real life all the time"*
		P14, F, 25	*"I would describe myself as quite critical of what I'm seeing and also aware that you can modify pictures in many different ways and it's not all real"*
		P15, F, 21	*"I'm quite a rational person, I can imagine it would affect some people quite badly but I would say I'm quite rational, it's just helped me to want to go the gym when I've not had motivation to go, it gives me motivation to go*
	Filtering and choosing relevant content to follow	P19, F, 22	*"I always like to follow normal people as well […] these things are actually achievable"*
		P15, F, 22	*When they put up their personal life and things…I'm not interested in them as a person which is probably quite bad but I just want to see the videos of what they do in the gym"*
